# Dentinal Dysplasia Type I: A Case Report with a 6-Year Followup

**DOI:** 10.1155/2013/659084

**Published:** 2013-05-14

**Authors:** Sezin Ozer, Bora Ozden, Feyza Otan Ozden, Kaan Gunduz

**Affiliations:** ^1^Department of Pediatric Dentistry, Faculty of Dentistry, Ondokuz Mayıs University, Atakum, 55139 Samsun, Turkey; ^2^Department of Oral and Maxillofacial Surgery, Faculty of Dentistry, Ondokuz Mayıs University, 55139 Samsun, Turkey; ^3^Department of Periodontology, Faculty of Dentistry, Ondokuz Mayıs University, Kurupelit, 55139 Samsun, Turkey; ^4^Department of Oral Diagnosis and Radiology, Faculty of Dentistry, Ondokuz Mayıs University, Kurupelit, 55139 Samsun, Turkey

## Abstract

*Introduction*. Dentin dysplasia is a rare disturbance of dentin formation characterized by normal enamel but atypical dentin formation with abnormal pulpal morphology that is inherited as an autosomal pulpal morphology. *Case Presentation*. A 7-year-old female who had problems in chewing function was referred to Oral and Maxillofacial Surgery Department at the Faculty of Dentistry in Ondokuz Mayıs University. In the radiographic examination, it was determined that some of the unerupted permanent teeth of the patient had short, blunted, and malformed roots with obliterated pulp chambers, although the bone below the teeth showed well-defined margins. This unusual case of generalized short roots presents a case demonstrating both classic and atypical features of dentinal dysplasia type I (DDI) in the mixed and permanent dentitions. *Conclusion*. There are still many issues in the diagnosis and management of patients with dentin dysplasia. Early diagnosis, clinical and radiographic findings, as well as treatment of this condition and the initiation of effective preventive strategies may help prevent or delay loss of dentition.

## 1. Introduction

Dentin is a mineralized tissue constituting the body of a tooth, serving as a protective covering for the pulp and as a support for overlying enamel and cementum [1]. Dentin dysplasia is an autosomal-dominant trait, affecting either the primary or both the primary and secondary dentitions for approximately one patient in every 100.000 [1, 2]. Dentinal dysplasia (DD) is classified into type I (DDI; radicular dysplasia) and type II (DDII; coronal dysplasia) in order to indicate the parts of the primarily involved teeth which affect both dentitions [2].

DDI is characterized by the presence of primary and permanent teeth with normal morphology of crown, but without rudimentary root development, incomplete or total obliteration of the pulp chamber, and periapical radiolucent areas or cysts [3, 4]. In DDI, teeth eruption is usually normal, but a delayed dental eruption pattern has also been reported [5]. Teeth are more resistant to dental caries, and clinically normal attrition has been found in both dentitions [6]. Because of short roots, DDI may be the extreme mobility and premature exfoliation of teeth, either spontaneously or with minor trauma [1]. 

Histopathologically, the enamel and the immediately subjacent dentin appear normal. Deeper layers of dentin show atypical tubular patterns with amorphous, atubular areas, and irregular organization. On the pulpal side of the normal appearing mantle of dentin, globular or nodular masses of abnormal dentin are seen. Dysplastic areas have been shown to exhibit tubules which are blocked and shunted from their normal course by numerous denticles [7, 8].

For DDI, treatment aims to remove sources of infection or pain, restore aesthetics, and protect posterior teeth from wear. Treatment varies according the age of the patient, severity of the problem, and presenting complaint [4]. However, the goal of the treatment is to retain the teeth for as long as possible. Because of the shortened roots, the prognosis for prolonged tooth retention is poor. When the teeth were lost prematurely, prosthetic replacement including dentures, overdentures, partial dentures, and/or dental implants may be required [9]. 

This paper describes a case of DDI in a 7-year-old girl, highlighting the clinical and radiographic variations of the defect and the importance of the preventive care in this anomaly. 

## 2. Case Presentation

A 7-year-old female was referred to the Department of Oral and Maxillofacial Surgery at Ondokuz Mayıs University, Faculty of Dentistry. The patient had some complaints in chewing function. The patient's medical history revealed chronic renal insufficiency and hypertension and received peritoneal dialysis five times a day. 

The extraoral examination revealed no abnormality. The intraoral examination revealed that the patients' oral hygiene was poor, and there were plaque deposits present in all quadrants. Only the maxillary left first primary molar had caries. The patient was in the mixed dentition, and the first permanent molars showed abnormal occlusal morphology. And the patient had class 2 malocclusion with anterior open bite. 

**Figure 1:**
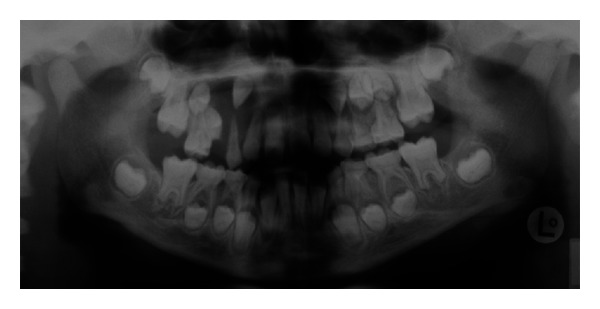
Panoramic radiographic view of the patient at first visit.

In the radiographic examination, there was a radiolucent cystic lesion at right maxilla, first permanent molars and incisors were erupted, and the root development was incomplete. The permanent second molars were unerupted and had no root formation, while the other unerupted permanent teeth had short, blunted, and malformed roots with obliterated pulp chambers, and bone below the teeth showed well-defined margins (Figure 1). 

Treatment strategy consisted of instructing the patient in oral hygiene and provision of treatment to enhance mastication and improve esthetics. Treatment was initiated with compomer restoration of the maxillary first primary molar. Then, dietary and oral hygiene instructions as well as fluoride supplements were suggested to the patient. The primary teeth had no mobility and infection, so they were not extracted. The possible complications were explained to the patient and her family, and then the patient was followed up. Depending upon clinical and radiographic examinations, the patient was diagnosed with DDI. Family history revealed no such abnormality among other family members. 

During the controls, the primary teeth of the patient were physiologically lost. The permanent maxillary right second premolar, the mandibular left first premolar, the mandibular third molars, and the second molars were extracted due to extensive mobility. However, the patients' oral hygiene was good, and the gingiva was healthy. 

**Figure 2:**
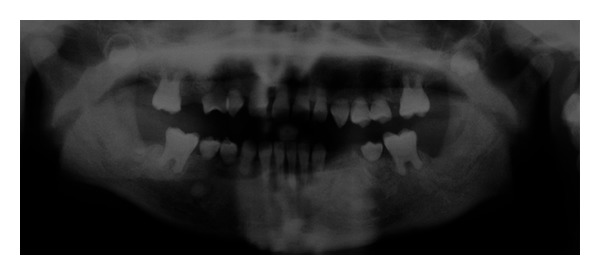
Panoramic radiographic view of the patient at last visit.

After six years, the permanent teeth were still in the mouth, and the patients' oral hygiene was still good. Complete obliteration of pulp chambers was noted in both erupted permanent teeth, and also they had no mobility, they were healthy and had no caries (Figure 2).

In this case, because of patient's age, dental implants and the other prosthetic management strategies were not suggested. The patient is still followed up. 

## 3. Discussion

DDI is a genetic defect of dentin formation, although its etiology is still considered a mystery [1]. Wesley et al. proposed that the condition is caused by an abnormal interaction of odontoblasts with ameloblasts leading to abnormal differentiation and/or function of the ectomesenchymal-derived odontoblasts [8, 10]. DDI should be differentiated from DDII, dentinogenesis imperfect, and odontodysplasia [11]. The present case exhibited features of DDI defined in the study, including clinically normal crowns, radiographic obliteration of pulp chambers, rootless teeth, and cystic lesion. Malocclusion is not a specific feature of DDI, but a few cases of malocclusion have been reported in association with this disorder [12]. In this case, class 2 malocclusion with anterior open bite was observed. 

DD is usually an autosomal dominant condition [1], and there is a 50% chance that a child born to an affected parent will himself be affected [9]. But, in this patient, there was no familial history of the disease, so she was considered to be the first generation sufferer. Some association has also been reported between dentine dysplasia and osseous chances in addition to sclerotic bone formation [13], but this patient had no signs of other pathologic conditions.

Management of patients with DD has presented dentists with many problems. Extraction has been suggested as a treatment alternative for teeth with pulp necrosis and periapical abscess. Endodontic treatment is contraindicated in teeth with total obliteration of root canals and pulp chambers [12]. Tidwell and Cunningham in 1979 concluded that the results of endodontic treatment might not be successful, but though challenging; they provided an alternative to extraction [6]. Orthodontic treatment is suggested; however, further resorption of the roots, loosening of teeth, and premature exfoliation may occur due to the resistance of the short roots to the orthodontic forces [14]. However, followup and routine conservative treatment is another important choice of treatment [15]. In DDI, because of the shortened roots, early tooth loosing from periodontitis is frequent [9]. For the maintenance of periodontal health of the mobile teeth, oral hygiene must be established and maintained. In this case, followup and routine conservative treatment plan was preferred for the patient, because the teeth had no mobility and the patient's oral hygiene was good. However, because of the early age of the patient, dental implants and the other prosthetic management strategies were not suggested. The patient is still followed up. 

## 4. Conclusion

DDI is an unusual abnormality of dentin which leads premature exfoliation of the primary and permanent teeth. The treatment for children with DDI mainly aims effective preventive care because the early loss of the teeth from periodontitis is frequent. The oral hygiene of the children must be established to have natural teeth as long as possible. The pediatric dentist has an important role in the early diagnosis of this disorder in guiding patient to prolong the retention of affected teeth.
